# An emerging artificial nanomachine: a nanoengine with a reversible clutch

**DOI:** 10.1038/s41392-024-01919-9

**Published:** 2024-08-26

**Authors:** Ziqi Fang, Jianxin Jiang, Min Wu

**Affiliations:** 1 School of Medical Information Engineering, Gannan Medical University, Ganzhou, China; 2 Wenzhou Institute, University of Chinese Academy of Sciences, Wenzhou, China; 3 Wound Trauma Medical Center, State Key Laboratory of Trauma, Burns and Combined Injury, Daping Hospital, Army Medical University, Chongqing, China

**Keywords:** Nanobiotechnology, Nanobiotechnology

Recently, a paper published in *Nature Nanotechnology*,^1^ describes a magnetically driven spherical rotating nanomotor with a reversible clutch system, capable of precisely regulating the force transmission between the engine and the driving element. This innovative design offers a fresh perspective for artificial nanomachines to exert mechanical functions within organisms, and fully demonstrates its potential as a nanomechanical machine in biomedicine.

Back in ancient times, rotation was thought to be a human creation, and the anatomy of living things likely indicates that rotational motion cannot exist in living organisms. Until 1973, the whirling motion of the flagella in *E. coli* reveals the existence of functional whirling in organisms, greatly challenging traditional notions. On this basis, the concept of the molecular motor was proposed. As a highly complex protein assembly, molecular motors can drive the structure to move in a straight line or rotation. Beyond bacterial flagella, myosin, kinesin, and dynamin are among the members of the molecular motor family within living organisms.^2^ These biological structures feature a clutch mechanism that temporarily dissociates the rotor from the engine’s force, enabling fine-tuning of its activity. However, in the current frontier of nanotechnology, replicating such intricate and precise mechanisms remains challenging. Because traditional nanomachines lack a clutchlike structure, their engine systems are always connected to the rotor, and the continuous force transmission limits their adaptability and accuracy, making them unable to meet the high standards of biological motors.^3^ This obstacle poses a major challenge to integrating artificial nanomachines into living systems.

**Fig. 1:**
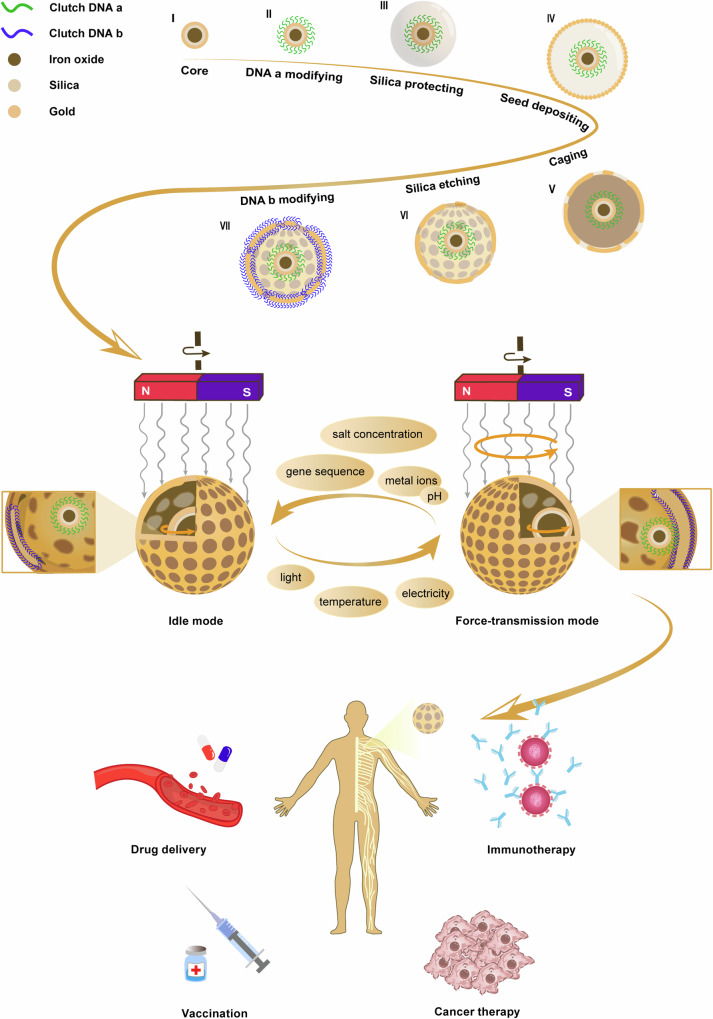
**Preparation, operation, and application of the golden cage spherical nanomotor**. The nanomotor uses iron oxide@silica@gold particle (I) as the engine, and the surface is modified with clutch DNA a (II). Then, using amorphous silica as a spherical template (III), the porous gold structure is formed on the surface of the silica template through synergistic mechanisms of gold seed-mediated growth (IV) and coalescence driving cage (V). After the removal of the silica template (VI), a thin and porous cage with crystalline nanopore surfaces is formed. The surface of the cage is modified with clutch DNA b (VII), resulting in a highly durable cage structure consisting of single crystal particles. The entire system is driven by a torque force and can be regulated by a variety of factors, including genetic sequence, salt concentration, metal ions, pH, light, temperature, electricity, etc. When the motor is in idle mode, the engine and rotor do not contact and the nanomotor does not work; when the motor is in force the transfer mode, the engine hybridizes with the DNA on the rotor, transferring the power of the engine to the rotor, and the nanomotors work. This system has the potential to be applied in biomedical fields, bringing new possibilities for drug delivery, cancer treatment, immunotherapy, and vaccination

In terms of the field of nanomachines, there are few reports on artificial nanomachine clutches, so a nanomachine with a reversible clutch is very attractive. Previously, There were precedents for using DNA in the nanosphere^4^ and for using magnetic actuators to regulate the movement of nanomachines.^5^ Lin et al. applied these two innovations to nanoengines, modifying the DNA of magnetic engines and spherical rotors to change the state of DNA-complementary nucleic acid hybridization by controlling the microenvironment, enabling adjustable assembly, and disassembly of DNA engines and rotors (like turning on and turn off) in a magnetic environment, thus controlling the state of the motor (Fig. 1). This innovative approach achieves precise control of nanomotors to a certain extent, not only avoiding the cost problem of irreversible DNA nanostructures, but also solving daunting challenges such as the inability to achieve cyclic operation.

Lin and his research team introduced the two states of the nanomotor in the paper, and demonstrated its synthesis steps and structural characteristics. They centered on iron oxide@silica@gold particle and used clutch DNA a for surface modification. Using silica as a spherical template, the porous gold structure was formed on the surface through gold seed-mediated growth and coalescence cage. After the template was removed, the clutch DNA b was used to modify the nanoengine to form a highly uniform cage structure (Fig. 1).^1^ The structure not only limits the movement of particles, but also can exchange with the microenvironment through pores. Therefore, the state between the engine and the rotor can be changed by controlling the microenvironment, and the working state of the nanomotor can be controlled. To study the reversibility of nanomachines, they adjusted the concentration of NaCl in the microenvironment. At higher concentrations, DNA hybridization accelerated, prompting the engine to transmit force to the rotor, and the nanomotors entered a force-transmission mode (turn on). Conversely, at lower concentrations, clutch DNA a and clutch DNA b separate, the engine does not contact the rotor, and the nanomotor returns to its initial idle mode (turn off). In addition to the salt concentration, there are many ways to affect the stimulation response system of DNA, such as gene sequence, metal ions, pH, light, temperature, electricity, etc. These ways greatly enhance the biocompatibility of nanomotors and demonstrate the broad application potential of controllable nanomotors.

Moreover, Lin et al. used the SLB platform as a tool to build a single particle rotation dynamic monitoring system, limiting the motion of a single particle to a two-dimensional plane, thus successfully solving the problem that is difficult to detect due to the small particle diameter and random Brownian motion. In order to verify the response and function of nanomotors to specific microenvironmental stimuli in a biological environment, the authors used microRNA (miRNA) as a model for environmental nucleic acids. By making different DNA modifications to the nanomotors, they achieved mechanical control of transgenic Notch signaling and natural integrins, and detected activation of different receptors using nuclear localization mCherry expression. The results showed that the activation of Notch and natural integrin was significantly increased under the combined action of nanomotors and miRNA. This finding strongly demonstrates that the designed nanoengines can bind to biological entities and act accordingly by activating mechanoreceptors in the organism. Given that miRNAs are widely recognized as effective biomarkers for the early diagnosis and treatment of tumors, the development of this system brings potential value to the field of early diagnosis and treatment.

In summary, Lin and colleagues crafted a magnetically driven rotating nanomotor with a reversible “clutch” system. They cleverly used the precise recognition ability of DNA to achieve mode conversion of the engine, breaking the bottleneck of static DNA nanotechnology.^1^ Further, the gold cage nanospace and magnetic wireless remote drive methods constructed in this study not only ensure the mechanical movement of nanomotors in space with low variability, but also lay a solid foundation for the wide application of nanomotors in organisms. Through experimental verification, they demonstrated that the structure has quite good biocompatibility and the feasibility of being able to interact with biological structures. These properties suggest broad applications in the biomedical field. By carrying out different DNA modifications, the nanomotor is expected to achieve targeted delivery of drugs, and the presence of a clutch structure makes it possible to release and stop drugs at a person’s will. When the target is a miRNA, the system can become a tool for precise early diagnosis and treatment of cancer. Not only that, the nanomotor can also play a role in vaccination as a delivery vehicle for the currently favorite mRNA vaccines. Similarly, by replacing the delivered substance with T cells or antibodies, the system can also be used for immunotherapy (Fig. 1). With the continued development of nanotechnology in medical and other fields, it is believed that the system will have ever-growing applications in the future.
